# Impact of a Task-Grabbing System for surgical technicians on operating room efficiency

**DOI:** 10.1038/s41598-024-54524-9

**Published:** 2024-02-21

**Authors:** Xiuwen Chen, Jiqun He, Luofang Peng, Li Lin, Pengfei Cheng, Yao Xiao, Shiqing Liu

**Affiliations:** 1 Teaching and Research Section of Clinical Nursing, Department of Operating Room, Xiangya Hospital, Central South University, Changsha, China; 2 Xiangya School of Nursing, Central South University, Changsha, China; 3 School of Business, Hunan University of Science and Technology, Xiangtan, China; 4 Department of General Surgery, Xiangya Hospital, Central South University, Changsha, China; 5 Department of Respiratory Medicine, Xiangya Hospital, Central South University, Changsha, China; 6 National Clinical Research Center for Geriatric Disorders, Xiangya Hospital, Central South University, Changsha, China

**Keywords:** Task-Grabbing System, Operating room efficiency, Quality improvement, Surgical technicians management, Outcomes research, Health care, Health services

## Abstract

The purpose of this study was to evaluate the effect of the Task-Grabbing System on operating room efficiency. Based on the competition-driven concept of the ‘Uber’ app, an Task-Grabbing System was designed for task allocation and quality assessment. We implemented the Task-Grabbing System in our hospital operating room and compared the differences in consecutive operation preparation time, turnover time, and task completion time performed by surgical technicians for tasks such as patient pick-up, operating room cleaning, medical equipment recovery, three-piece set delivery, as well as blood gas analysis and intraoperative specimen submission before (October 2019) and after (December 2019) the implementation of the Task-Grabbing System. After the implementation of the Task-Grabbing System, the consecutive operation preparation time was reduced from the average of 43.56–38.55 min (*P* < 0.05), and the turnover time was decreased from the average of 14.25–12.61 min (*P* < 0.05). And the respective time consuming of surgical technicians for patients picking up, operating room cleaning, medical facilities recovering, the three-piece set delivering, blood gas analysis sending and intraoperative specimen submitting was significantly shortened (*P* < 0.05). The Task-Grabbing System could improve the operating room efficiency and effectively mobilize the enthusiasm and initiative of the surgical technicians.

## Introduction

Providing surgical treatment for patients, the operating room always makes overwhelming contributions to and figures prominently in the thriving and forging for a hospital running^1–3^. With the number of surgeries continuously increasing and the difficulty degree of those deepening, the optimization of relevant procedures in operating room and the management upon quality and efficiency have become the key issues of the hospital management^4–7^. Operating tasks are complicated, which require the highly efficient joined efforts of surgeons, operating room nurses, anesthesiologists, and surgical technicians, from pre-operative patients preparing, patients picking up, anesthesia preparing, medical facilities delivering, to intraoperative pathological specimen sending, blood gas analysis of examination submitting, blood taking from blood bank, post-operative transferring, post-operative room cleaning up, etc. Any delay in any part of the chain above will influence the efficiency and quality of the operation, and thus affect the turnover rate of patients in the operating room^3,7–10^.

Assuming responsibilities for patient transfers, operating room cleaning, medical equipment delivery, and the retrieval of relevant instruments, surgical technicians play a significant role in the surgical cycle chain^11–13^. Currently, in China, information transmission and collaboration among different departments during surgical procedures are primarily conducted via telephone. When surgical technicians are required during ongoing surgeries, circulating nurses relay the requirements to logistics staff by phone, who in turn assign tasks to surgical technicians over the phone. However, under this mode medical staff can often suffer from busy telephone lines, incorrect information transmission, failure in real-time tracking, and low enthusiasm of surgical technicians actuated by merely accepting the tasks passively, which give rise to delays in patients transferring, the post-operative operating room cleaning, and medical facilities dispatching. Such delays or errors in delivery occur from time to time, which have already considerably affected the work efficiency and brought many security risks^9,14,15^.

To improve operating room efficiency, recent research has concentrated on the development of sophisticated information systems. For instance, Nouei et al.^16^ created an integrated system using Kinect sensors and RFID technology, enabling touchless interaction and automated data recording. Their research emphasized the significance of streamlined workflow integration, maximizing the performance of surgical teams. Likewise, Brown et al.^9^ introduced a coordinated patient transport system, leading to a substantial reduction in idle operating room time and a fourfold improvement in on-time starts, highlighting the effectiveness of technology-based solutions in enhancing hospital efficiency. In line with these efforts, our hospital has developed a software for task allocation and real-time information retrieval during surgery. Derived from logistics distribution and traffic informationization, such as "Uber”, tasks grabbing was an tasks allocation model based on information interaction and the competitive notion of priority access to information under the Android platform. Our study aimed to assess the efficiency of operating room cooperation and the motivation of surgical technicians before and after implementing the Intelligent Task-Grabbing System through a prospective controlled study.

## Methods

### Study population

Our hospital is an officially acknowledged large comprehensive Level 3 Grade A Hospital with 3500 approved beds, 55 operating rooms, and an average monthly operation volume of over 6000. A total of 48 surgical technicians worked in the operating room, including 17 males and 31 females; aged 21–58 with an average age of 46.2 years; 3 with college degrees, 19 with high school diplomas, 22 with junior high school education, and 4 with primary school education; Their lengths of service ranged from 6 to 147 months, with an average of 37.8 months. It has been estimated that surgical technicians receive a total of about 1100 tasks per day, mainly for operating room cleaning, patients picking up, intraoperative pathological specimen inspecting, medical equipment delivering, instruments recovering, three-piece sets (beds, breathing balloons, oxygen bags) dispatching, and locker room managing, etc.

### Traditional management mode

#### Position assignments

Implemented a position responsibility management system, in which 48 surgical technicians were assigned to 7 different position and each position was reasonably arranged according to the workloads, involving 3 management positions (responsible supervisor, squad leader and general dispatcher, each 1), 12 in the field, 17 in the back office (12 in cleaning, 5 in full-time plane hygiene), 4 in logistics, 4 in distribution, 3 in night shifts, and 3 in locker rooms. With the 8-h work system continuous or overlapping shifts were hinged on the operating hours. And two additional staff members were designated for flexible, mobile shifts.

#### Position responsibilities

The responsible supervisor was responsible for the overall tasks; the squad leader took charge of staff training, quality inspection and partial general scheduling; the general dispatcher was appointed to dispatch and record the work of receiving patients, sending specimens, and cleaning and sanitizing in the operating room; the field service personnel worked for external auxiliary assignment, including transporting patients, sending fast pathological specimens for examination, and taking blood, etc. keeping the operating room and auxiliary room was mainly carried out by the staff in back office; logistics was required to ensure the recovery of equipment, cleaning, packing, and finishing of some precise and valuable instruments, as well as cleaning of the logistics area; Distribution department was in charge of preparing surgical instrument packs, distributing surgical supplies, and transporting transfer beds; Night shifts focused on all auxiliary tasks related to emergency surgery, including transferring patients, operating room cleaning, and goods delivering; locker room administrators were obliged to do access management for medical staff, maintain the changing room clean and tidy, receive and distribute clean surgical clothes, wash surgical shoes, etc. In addition to arranging 3 surgical technicians for medical treatment on weekends, other personnel participated in the wholly cleaning of the operating room.

#### Task receiving management

Included two types fixed tasks were completed according to the corresponding process or the preset time, such as aisle cleaning, locker room managing, etc. Additionally, other randomized tasks were informed by telephone or performed through surgical technicians’ independent inspection and receiving instructions from nurses, like patients picking up, contaminated equipment recycling and inter-operative rooms cleaning up. Telephone notification means that the general dispatcher will call other employees to assign tasks upon receiving instructions from the circulating nurses. For independent inspections, employees in each post will check the progress of the operating room in the area of which they take charge themselves. Once the operation is over, they will perform tasks consciously, mainly referring to cleaning and sanitation in the operating room and recycling of contaminated materials.

### The Task-Grabbing System management mode

Position assignments and responsibilities were consistent with traditional model management, while tasks receiving management was conducted by the grabbing software and hardware, The Task-Grabbing System consisted of the following modules: cooperative task assignment module, mobile task reception, data analytics, and configuration settings.

#### Cooperative task module design

Tailored to meet clinical needs and based on the roles of surgical technicians, keywords were set as buttons for task selection, such as patients picking up, patients delivering, blood taking from blood bank, medical facilities collecting, inter-operative cleaning, blood gas analysis specimens sending, intraoperative pathological specimen sending, tasks checking, tracking, evaluating, and so on. The menus are arranged which could be displayed on the tablet computer interface for the circulating nurses using in the operating room to send tasks.

#### Mobile terminal module design

Commence the installation of 'Medical Manager' app version 5.2.1.4861 on the mobile devices used by surgical technicians. The app fully takes the employees' working rights, scheduling time, real-time query and data statistics into account. There are four major core functions in the APP interface: *homepage*, *tasks grabbing*, *to-do lists*, and t*he personal center*. The *homepage* function area allows for tasks such as sign-in, equipment information queries, delivery tracking, personal workload inquiries, and problem or fault reporting; *the tasks grabbing* is the order-taking interface for various tasks, where the surgical technicians can select the task by themselves after receiving the instructions from circulating nurses; in the *to-do lists* function area, the unfinished tasks can be viewed in real time; *the personal center* displays individual general information, including name, work number, phone number, email, and also allows for account logout.

#### Hardware preparation and installation

The system required five parts to run the hardware environment: (1) Suspended tablet computer. Installed “Medical Manager”, an information system for tasks grabbing. The tablet computers were equipped in each operating room, for nurses to send instructions. (2) Smartphone. Also installed “Medical Manager” order system for surgical technicians’ grabbing. (3) Bluetooth-enabled tracking system. Set up at each task executed area (installed at the automatic door in the operating room), it was used to record the time and frequency of medical assistants’ arriving. (4) Computer. It was set up at the nurse station for the manager or the general dispatcher to understand the holistic conditions of order grabbing as well as instruction executing and to get command of the overall monitoring for the system's operation. Besides, with computer the manager can dispatch orders manually in case of special situations. (5) Network environment. The operating room is equipped with 4G network coverage.

#### Process

**Figure 1:**
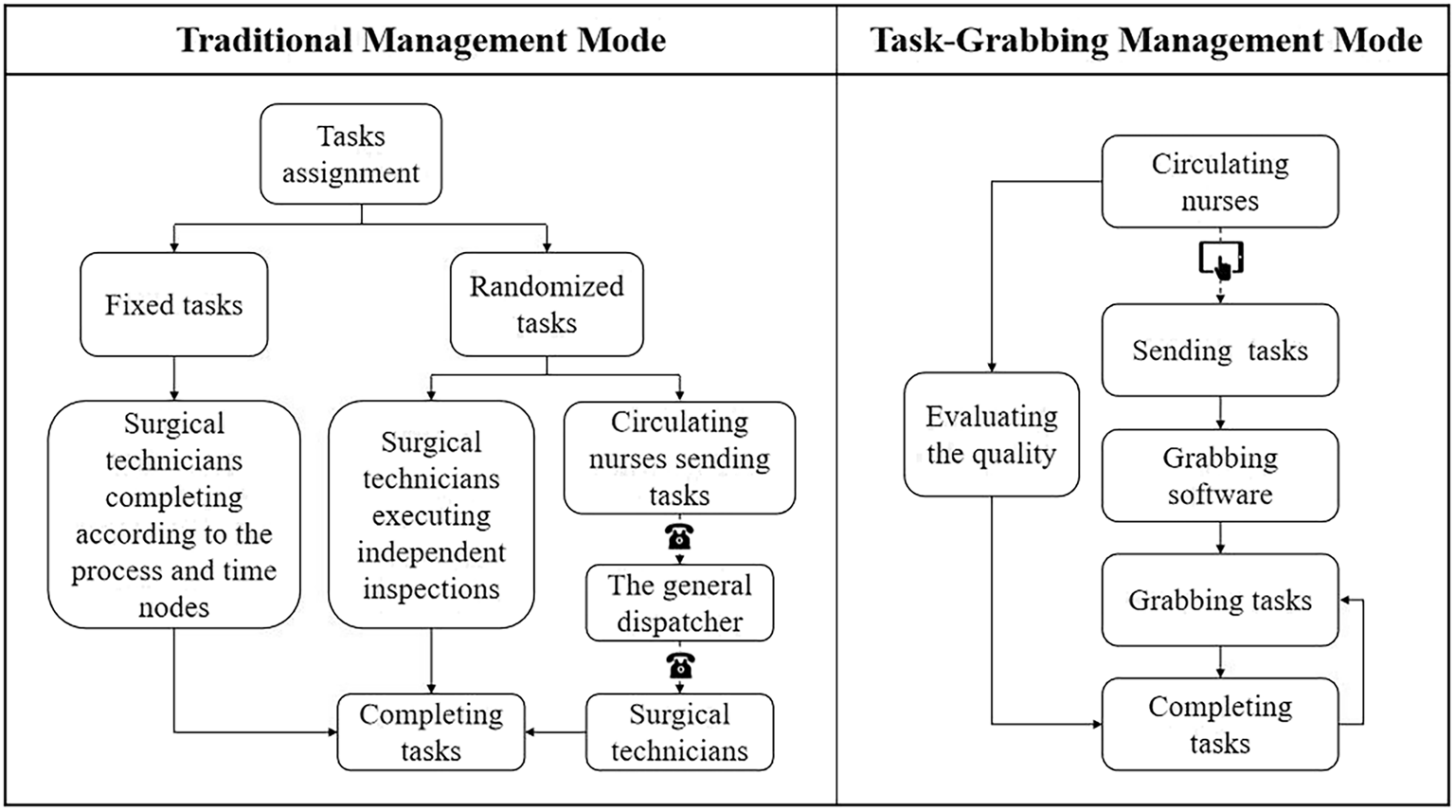
Diagrams of the traditional management mode and the Task-Grabbing System management mode.

Take the cleaning of the operating room as an example. The whole process was divided into 6 steps: Step 1. Send instructions. When the patient leaves the operating room after the operation, the circulating nurse clicks the “clean” button on the tablet. Step 2. Grab the tasks. The mobile phone of the surgical technicians who have the corresponding authority (having received the standard training and on their shifts) will sound the alarm as “tik tik tik…”. If there is no ongoing task, they can click to grab the task. Contrarily if there are unfinished tasks at hand, they cannot grab orders. Step 3. Arrive at the operating room and the start time will be recorded. The employee who has successfully grabbed the order arrives at the designated operating room with a cleaning vehicle (with sanitary tools), and the employee's arrival time can be automatically recorded through the Bluetooth-enabled tracking system at the automatic door of the operating room. Step 4. Perform the task. Clean and sanitize the operating room according to the standard cleaning procedures. Step 5. After the surgical technicians complete the cleaning work, they will take the sanitation vehicle out of the operating room and approached the Bluetooth-enabled tracking system to record the finishing time. Step 6. Evaluate the quality of the work. On the “Quality Evaluation” module in the task bar of the tablet computer, the circulating nurse ticks the corresponding options to make an overall evaluation of the quality the auxiliary’s work. Two management modes are graphically shown in Fig. 1.

### Data collection procedures

The consecutive operation preparation time and turnover time were the primary outcome measures of this study. The consecutive operation preparation time refers to the time interval from the last surgical patient leaving from the operating room till the next surgical patient's actually started the case, while the turnover time means the length from the last leaving till the new surgical patient entering the operating room, which can be recorded by video monitoring. Note that turnover time does not include all aspects of operating room cleaning, some of which were performed after the new patient entered the operating room.

The secondary outcome measures were the actual time required for the surgical technicians to complete the task: picking up the patient, cleaning the operating room, recovering medical equipment, sending a three-piece set, submitting blood gas analysis and examination, taking blood from blood bank, and dispatching the intraoperative specimen. These data were collected by circulating nurses using the unified form to fill in and record the start and the end of the time. The starting time of the control group was the time to call the first task assignment through telephone, while the starting time of the experimental group was the task-sending time on the tablet computer, and the end time both referred to the time when the tasks were completed.

Finally, we compared the indicators collected before (October 2019) and after (December 2019) the implementation of the Task-Grabbing System, with November 2019 dedicated to system testing.

### Data analysis

The database was established with SPSS18.0 software for data collection and statistical analysis. The measurement data in the general information of the study objects were described by the average number, and absolute frequency as well as the percentage was adopted to present the enumeration data. The statistical significance was set at a *p* value less than 0.05 for all statistical tests, and testing was two-sided.The measurement data of the baseline information were compared by *χ*^2^ test or *t-*test. The comparisons with the main outcome indexes and the second outcome indexes were conducted by *t*-test.

### Quality control procedures

To get the data effectively collected, the subject team conducted training to all the faculty on form writing for the research; hired a full-time graduate student for supervision and data collection. A head nurse was appointed to regularly check the form filling and include it into quality management. Circulating nurses were assigned to their own specifically designated operating room and any minor discrepancies of circulating nurses’ length of their services, title and working experience was promised. Additionally, digital time clocks were installed in each operating room and precisely calibrated.

### Ethical approval

The experimental protocol was established, according to the ethical guidelines of the Helsinki Declaration and was approved by the Human Ethics Committee of Xiangya Hospital.

## Results

### Clinical characteristics

The experimental group had 4,217 operations and the control group had 4095 operations. Table 1 shows the respective data on procedure type, operation type, surgical position, anesthesia mode, urethral catheterization status, and gender of the surgical patients before and after the application of the Task-Grabbing System. The data in Table 1 indicate that there were no statistically significant differences except the gender of the surgical patients (*P* > 0.05).

**Table 1:** Surgical case distribution.

		Number of cases before the Task-Grabbing System (n = 4217)	Number of cases with the Task-Grabbing System (n = 4059)	*χ*^2^ or *t*	*P*
Procedure type	Open	2748	2597	2.473	0.119
	Laparoscopy	1469	1492		
Operation type	Emergency operation	542	503	0.398	0.530
	Scheduled operation	3675	3556		
Surgical position	Supine position	1898	1754	2.705	0.101
	None-supine position	2319	2305		
Anesthesia mode	General anesthesia	3874	3723	1.278	0.865
	Intrathecal anesthesia	71	67		
	Nerve blocking anesthesia	122	116		
	Local anesthesia	84	82		
	Extracorporeal circulation anesthesia	66	71		
Urethral catheterization status	Without catheterization	508	479	0.425	0.808
	Catheterized at the ward	1475	1401		
	Catheterized at the operating room	2234	2179		
Gender	Male	2024	1826	7.531	0.006
	Female	2193	2233		

### Clinical outcomes

Table 2 demonstrates that the average of the consecutive operation preparation time and turnover time after the implementation of the Task-Grabbing System were considerably shorter than before, and these differences were statistically significant (*P* < 0.05).

**Table 2:** Operating room efficiency data.

Group	Before the Task-Grabbing System	With the Task-Grabbing System	*t*	*P*
The total number of the operations	2392	2438		
The operation preparation time (Mean ± standard deviation) (min)	43.56 ± 4.575	38.55 ± 5.625	− 33.918	0.000
The turnover time (Mean ± standard deviation) (min)	14.25 ± 2.356	12.61 ± 2.424	− 23.847	0.000

Table 3 shows that the actual time spent by the surgical technicians to complete the patients picking up, operating room cleaning, medical equipment recovering, three-piece delivering, blood gas analysis sending and intra-operative specimen submitting of the Task-Grabbing System was significantly shorter than that of the control group (*P* < 0.05). However, there was no statistically significant difference in the time taken to complete the task of taking blood from the blood bank (*P* > 0.05).

**Table 3:** Completing tasks efficiency data comparison.

		Before the Task-Grabbing System	With the Task-Grabbing System	*t*	*P*
Patients picking up	Cases	2758	2655	− 35.771	0.000
	Time (min)	20.25 ± 3.654	23.68 ± 3.404		
Operating room cleaning	Cases	3518	3226	− 71.581	0.000
	Time (min)	8.81 ± 2.129	12.29 ± 1.857		
Medical equipment recovering	Cases	3703	3609	− 111.855	0.000
	Time (min)	22.51 ± 7.610	50.83 ± 13.339		
Three-piece sets delivering	Cases	4568	4494	− 28.991	0.000
	Time (min)	5.64 ± 2.164	6.85 ± 1.784		
Blood gas analysis sending	Cases	2160	2193	− 18.703	0.000
	Time (min)	14.94 ± 2.517	16.40 ± 2.639		
Blood taking	Cases	361	350	− 0.887	0.375
	Time (min)	16.20 ± 4.398	16.48 ± 4.199		
Intra-operative specimen submitting	Cases	1080	1110	− 2.329	0.020
	Time (min)	3.90 ± 1.880	4.11 ± 2.234		

## Discussion

Surgical technicians are an important part of the operating collaboration, and their performance can make a big difference on the efficiency of the operation^4,8,11^. Having not been professionally medical educated, and varying educational backgrounds, surgical technicians can be greatly difficult to manage. The drawbacks of the traditional management model were as follows: (1) Not timely. Due to the different speeds of surgeries conducting, some operating rooms changed swiftly, and thus cleaning and sanitizing work were accumulated, which could not be handled promptly by the staff working for other position responsibilities. (2) Waste of human resources. Regardless of the workload, the position responsibility managing system requires highly sufficient manpower equipped, otherwise it will affect the efficiency of consecutive surgeries. (3) Workers are often too passive and lack of motivation. The jobs are scattered, and it is quite difficult to count the workloads, to judge their performance, resulting in low initiative. (4) Dispatching can be hard to achieve. Under the traditional management mode, there are often inevitable issues such as busy telephone calls, incorrect dialing of phone numbers, incorrect transmission of information, and inability to track in real time. With the hospital information infrastructure development, it has seen the inexorable trend that the traditional management mode which was simply based on empirical cognition must be transformed to a modern informationizing and management mode. In this study, the design of the Task-Grabbing System closely adhered to current medical standards, notably the robotic standard development life cycle framework presented by Olszewska and colleagues^17^. This adherence encompassed various critical steps, including the identification of key concepts, a dedicated focus on their development and formalization, as well as the thorough validation and practical application in real-world scenarios. Our hospital has pioneered the implementation of an software information system for task allocation management within the logistic support department of the operating room. This pioneering initiative represents an innovative approach to order management for surgical technicians, marking a significant advancement in our operational procedures.

As a significant department of the hospital, the operating room is a unit with abundant medical resources, the continuously improving efficiency of which plays a crucial part in accelerating the turnover of surgical patients and increasing the economic and social benefits^18–21^. Therefore,the work efficiency of the operating room has already become an overwhelming indicator evaluating the quality of surgical care. Studies such as Weld et al. pointed out that the estimated cost of the operating room was $ 15 per minute, which accounted for about 40% of hospital revenue, and opportunity cost per operating room min was $9–$26^8,22,23^. The results of this study demonstrated that the adoption of the Task-Grabbing System led to a reduction in the average consecutive operation preparation time, from 43.56 to 38.55 min, and a decrease in the turnover time between consecutive operations, from an average of 14.25–12.61 min. The differences mentioned above were statistically significant (*P* < 0.05), which revealed that the efficient operation of the Task-Grabbing system could reduce the consecutive operation preparation time and turnover time. Under the busy situation with an average daily operation volume of 4 to 7 operations in each operation room of large hospitals, it is the consecutive operation preparation time and turnover time that really matter to the working efficiency of the operating room. And shortening the continuous operation time is a key measure to improve the efficiency. Between two consecutive operations, numerous tasks need to be accomplished, including transferring the last patient, cleaning the operating room, recovering medical equipment, distributing aseptic materials, and preparing for the next surgical patient. In our study, the Task-Grabbing System had transferred the traditional management mode into a task-driven initiative system, which effectively got rid of the shortcomings in the traditional management mode, and avoided delays in transferring of surgical patients, cleaning of the operating room, and delivering of medical equipment, successfully smoothed the connection between the operations and increased the working efficiency. Numerous studies have also indicated that information technology can significantly enhance surgical efficiency^2,9,21^. A systematic review by Bellini et al. determined that the use of artificial intelligence in the field of operating room organization could allow a more precise scheduling and limit the waste of operating room resources^2^. Brown et al. designed a coordinated patient transport system (CPTS) for ICU patients requiring surgery and unfolded that the implementation of CPTS resulted in a fourfold improvement in on-time operating room starts while significantly reducing idle operating room time^9^. Weld et al. has revealed that the use of Team STEPPS could diminish the average operation time by 12.7 min, and the incidence of patients’ safety problems has additionally decreased from the original 16–6%, which not only advanced the surgical efficiency, but also dwindled the occurrence of patients’ safety problems^8^. Julian et al. developed a computer-based tutoring system to train the cognitive and procedural surgery skills^24^. It is evident that hospital administrators can enhance the efficiency of operating rooms through the use of technology-driven systems.

Moreover, as shown in the results, the Task-Grabbing System has successfully reduced the time required for surgical technicians to complete tasks of receiving patients, cleaning the room, recycling equipment, sending three-piece sets, submitting the blood gas analysis and intraoperative specimen, indicating that this mode of management could effectively improve the timeliness of the task execution. The related factors could be analyzed as following parts. (1) The Task-Grabbing System can boost the enthusiasm of surgical technicians. Under the traditional mode of management, the surgical technicians completed the fixed tasks, by which the technicians were assigned to work at the fixed operation room, leading to the scattered position situation and difficulty in workloads counting. Given their relatively advanced age and limited educational backgrounds, improving their work initiative through training alone is challenging. General issues of negligence and procrastination cannot be overlooked. The Task-Grabbing System can automatically record the task details and workload of each employee participating in the bidding process, and it can automatically generate reports at the end of the month. This enables managers to quantitatively assess employee workloads. (2) The Task-Grabbing System can solve the problems such as busy telephone lines and incorrect information transmission caused by the demerit of the traditional management mode. Previously when an operating room needs to send the instructions to auxiliaries, the circulating nurse should first call the general dispatch center, who then should pass the call to the corresponding person in charge. Multi-party phone transfers often induce busy calls, wrong phone numbers and misunderstanding of the information, which tremendously affect the accuracy and timeliness for task executing. With the Task-Grabbing System, when the circulating nurse sends a task via the tablet computer, surgical technicians can promptly access it on their smartphones. The task's specific content and precise location are clearly and concisely displayed on the technicians' phones, allowing for discreet review.

It is worth noting that after the implementation of the Task-Grabbing System, the length of medical facilities recovering was shortened from an average of 50.83–22.51 min. Under the traditional management mode, as for the equipment recovery, the appointed surgical technicians are obliged to collect all the reusable equipment at a fixed time for disinfection. However, the Task-Grabbing System allows the technicians to conduct immediate response and immediate execution. Each task can be traced and queried, which not only ensures the timely recovery of dirt equipment, but also promotes the supervision of that. Additionally, the timely recovery of the medical instruments not only increases the turnover rate and save costs but also contributes to instrument maintenance, reducing the risks of wear and damage while helping control infection^25^. Prolonged exposure of contaminated instruments to a polluted operating room increased the risk of infection^26,27^.

By implementing the scientific management for surgical technicians, which includes tasks assignment, quality control, quality evaluation and workloads counting etc., the Task-Grabbing System has boosted the enthusiasm and initiative of medical auxiliaries. It has significantly improved the efficiency of transitioning between consecutive operations without increasing the workforce, making it a highly valuable system that deserves wider adoption and promotion.

While this study has provided valuable insights, it is essential to acknowledge its limitations. Firstly, the Task-Grabbing System was exclusively implemented in a single hospital, which may have influenced the outcomes due to the institution's specific management style, workflow, and unique factors. Secondly, it is important to note that this study does not follow the rigorous design of a randomized controlled trial. Although we have employed robust methodologies for data collection and analysis, the absence of randomization may introduce certain biases or confounding variables that could affect the study's internal validity. In the future, a multicenter randomized controlled trial could be considered to validate the findings of this study.

## Conclusions

In conclusion, this study demonstrated that the implementation of the Task-Grabbing System significantly reduced consecutive operation preparation time, turnover time, and task-completing time. The findings indicate that the Task-Grabbing System is an effective strategy for hospitals to enhance operating room efficiency and stimulate the enthusiasm and initiative of surgical technicians. This system warrants clinical recommendation.

## Data Availability

The datasets generated during and/or analyzed during the current study are available from the corresponding author on reasonable request.
