# Author Correction: Impact of a Task-Grabbing System for surgical technicians on operating room efficiency

**DOI:** 10.1038/s41598-024-64357-1

**Published:** 2024-06-12

**Authors:** Xiuwen Chen, Jiqun He, Luofang Peng, Li Lin, Pengfei Cheng, Yao Xiao, Shiqing Liu

**Affiliations:** 1 Teaching and Research Section of Clinical Nursing, Department of Operating Room, Xiangya Hospital, Central South University, Changsha, China; 2 Xiangya School of Nursing, Central South University, Changsha, China; 3 School of Business, Hunan University of Science and Technology, Xiangtan, China; 4 Department of General Surgery, Xiangya Hospital, Central South University, Changsha, China; 5 Department of Respiratory Medicine, Xiangya Hospital, Central South University, Changsha, China; 6 National Clinical Research Center for Geriatric Disorders, Xiangya Hospital, Central South University, Changsha, China

Correction to: *Scientific Reports* 10.1038/s41598-024-54524-9, published online 21 February 2024

The subsection headed “Data collection procedures” in the Methods section was incomplete. The following sentence has been added to the end of the subsection’s first paragraph:

“Note that turnover time does not include all aspects of operating room cleaning, some of which were performed after the new patient entered the operating room.”

In addition, the Data Availability section in the original version of this Article was incorrect. The section now reads:

“The datasets generated during and/or analyzed during the current study are available from the corresponding author on reasonable request.”

The original Article has been corrected.

